# The Book World of Medicine and Science

**Published:** 1898-05-21

**Authors:** 


### The Book World of Medicine and Science.

Diseases of the Upper Respiratory Tract: the Nose, Pharynx, and Larynx. By P. Watson Williams, M.D.Lond., Physician to Out-patients, and in charge of the Throat Department, at the Bristol Royal Infirmary. Third Edition. Illustrated. (Bristol: John Wright and Co., 1898.) Price 8s. 6d.

The speedy appearance of a third edition of this work shows better than anything which we can say would do that it has met a want, and that the author's treatment of his subject has received the approval of a large circle of readers. And, indeed, it is a book which well deserves such a form of appreciation, for it is admirably adapted for the purpose for which it was written, viz., to serve as a practical guide to students and practitioners in the study of diseases of the upper air passages, and as a stepping stone to larger and more comprehensive works for those who wish to pursue the subject further. One of the points which strike us at once on opening the book is the profuseness with which it is illustrated, and on examining these illustrations we cannot but admire, on the one hand, the aptness with which many even of the most sketchy of the woodcuts throw light upon the descriptions in the text; and, on the other hand, the great beauty, as representations of diseased conditions, of the numerous chromo-lithographs which are copied from original drawings from nature made by Dr. Watson Williams. The book begins by a very useful chapter on the anatomy and physiology of the parts concerned, then it describes the various symptoms met with, and gives an account of the methods of examination which have to be made use of. After this the various diseases are taken in detail, and lastly come a few pages of formulæ and a general index, making a goodly volume of 282 well-printed pages. We should describe this as in every way a most satisfactory book, and one which ought to satisfy the reader as to the necessity and advantage of specialism of the better kind. When specialism consists of the grafting of a special knowledge of the diseases of certain regions upon a wide general knowledge of disease at large, and when the specialist puts his knowledge at the disposal of his brethren, as is done by the author of this book, specialism shows itself in its most attractive light.

Llangammarch Wells as a Health Resort. By W. Black Jones, M.D., B.S.Lond., D.P.H. (London: J. M. Kronheim and Co. 1898.) Price Sixpence.

The object of this book is to make known the virtues of Llangammarch as a health resort, and we think none the worse of a book for thus openly declaring its purpose. For, indeed, who is to know of the opportunities which exist in our own land for the carrying on of bath treatment—what in other countries are called "cures"—unless the medical men who reside at our spas will be good enough to do as Dr. Black Jones has done in the little work before us, and give some account of the treatment which is available at them, and the sort of cases in which it is found most useful. From Dr. Black Jones' description we gather that Llangammarch is a small village in Breconshire, situated on a tributary of the Wye, reached in about seven hours from London, standing about 600 feet above the sea level, and having hills in the neighbourhood running up to an altitude of 1,560 feet. About a mile from this village are situated the Lake Hotel, pump house, baths, and mineral springs. The latter have this peculiarity, by which they are differentiated from most other springs, that they contain an appreciable amount of chloride of barium—between six and seven grains to the gallon. Lithium is also found. Dr. Black Jones enters fully into the question of the therapeutic effects of barium, but it is quite clear that there are at his disposal at Llangammarch Wells many other useful influences besides the water itself. The cases which he principally writes upon are those of scrofula, debility, anæmia, and heart disease. The treatment of the latter seems, from his account, to be made somewhat of a specialty, and to have been attended with good results. We cannot but feel that there is a large opening for the development near home of that sort of bath treatment which is so popular abroad, and we are glad to see from this little book that at Llangammarch Wells such a reliable barium spring is within easy reach.

Inflammation of the Bladder and Urinary Fever. By C. W. Mansell Moullin, M.D., F.R.C.S. (London: H. K. Lewis. 1898.) Pp. 153. Price 5s.

As Mr. Mansell Moullin remarks, in his preface to this book, it is not admitted everywhere as beyond dispute that inflammation of the bladder is always due to micro-organisms; that urinary fever is nothing but septic infection and intoxication occurring under special conditions; and that catheters are always septic unless they have been thoroughly sterilised. The figment of urinary fever from shock and vague "reflex" conditions has been maintained for years to shield dirtiness and carelessness. It should now be laid decently to rest. Mr. Moullin deals in this volume, succinctly and compendiously, with cystitis from the clinical, pathological, and bacteriological points of view. After alluding to the probability that absorption never takes place from the bladder if damage to the epithelium has not been sustained, Mr. Moullin points out that the uro-bacillus is the only organism which, by itself, is able to set up suppurative cystitis. The reason is that the bladder, unless its resistance is lowered by injury, is able to grapple with any organism that may occur in the urine. This is the explanation of Roberts' cases of bacteruria. But the uro-bacillus, by decomposing urea in the bladder, may set up irritation from the free ammonia, and hence produce an inflammation, in the presence of which suppuration can be easily brought about. Mr. Moullin regards "urinary fevers" as of four main types. (1) Simple rigors due to the absorption from urine of septic poisons; (2) grave conditions following operations on the urinary organs, due to entrance into the blood stream of overwhelming doses of toxines; (3) septic fever following the catheterisation of persons whose kidneys are diseased; (4) septic infection of operation wounds of urinary organs. The book is completed by accounts of the treatment of suppurative and tuberculous cystitis. We note that Mr. Moullin—following Guyon—advocates the free employment, in many conditions, of suprapubic drainage through incisions. This book is one which it will be to the advantage of most general practitioners to study. It deals with views and facts recognised in the best hospital practice, but which have not yet found their way into the text books. It is clearly and temperately argued, and no views are advanced which are not supported by a sufficiency of evidence.

